# Molecular pathways and nutrigenomic review of insulin resistance development in gestational diabetes mellitus

**DOI:** 10.3389/fnut.2023.1228703

**Published:** 2023-09-20

**Authors:** Patricia Guevara-Ramírez, Elius Paz-Cruz, Santiago Cadena-Ullauri, Viviana A. Ruiz-Pozo, Rafael Tamayo-Trujillo, Maria L. Felix, Daniel Simancas-Racines, Ana Karina Zambrano

**Affiliations:** ^1^Facultad de Ciencias de la Salud Eugenio Espejo, Centro de Investigación Genética y Genómica, Universidad UTE, Quito, Ecuador; ^2^Facultad de Ciencias de la Salud Eugenio Espejo, Universidad UTE, Quito, Ecuador; ^3^Centro de Investigación de Salud Pública y Epidemiología Clínica (CISPEC), Universidad UTE, Quito, Ecuador

**Keywords:** gestational diabetes mellitus, insulin resistance, nutrients, metabolic pathways, nutrigenomic

## Abstract

Gestational diabetes mellitus is a condition marked by raised blood sugar levels and insulin resistance that usually occurs during the second or third trimester of pregnancy. According to the World Health Organization, hyperglycemia affects 16.9% of pregnancies worldwide. Dietary changes are the primarily alternative treatment for gestational diabetes mellitus. This paper aims to perform an exhaustive overview of the interaction between diet, gene expression, and the metabolic pathways related to insulin resistance. The intake of foods rich in carbohydrates can influence the gene expression of glycolysis, as well as foods rich in fat, can disrupt the beta-oxidation and ketogenesis pathways. Furthermore, vitamins and minerals are related to inflammatory processes regulated by the TLR4/NF-κB and one carbon metabolic pathways. We indicate that diet regulated gene expression of *PPARα*, *NOS*, *CREB3L3*, *IRS*, and *CPT I*, altering cellular physiological mechanisms and thus increasing or decreasing the risk of gestational diabetes. The alteration of gene expression can cause inflammation, inhibition of fatty acid transport, or on the contrary help in the modulation of ketogenesis, improve insulin sensitivity, attenuate the effects of glucotoxicity, and others. Therefore, it is critical to comprehend the metabolic changes of pregnant women with gestational diabetes mellitus, to determine nutrients that help in the prevention and treatment of insulin resistance and its long-term consequences.

## Introduction

Gestational diabetes mellitus (GDM) is a condition marked by raised blood sugar levels (hyperglycemia) that first appears during gestation, mainly in the second or third trimester (1). According to World Health Organization (WHO), hyperglycemia in pregnancy affects 16.9% of pregnancies worldwide (2). Furthermore, GDM prevalence has been highly described in Southeast Asia (20.8%), the North Africa and Middle East (27.6%) (3).

The diagnostic values for GDM can vary based on the healthcare guidelines used. However, the following blood glucose values are commonly associated with gestational diabetes: fasting blood glucose: ≥5.1 mmol/L; 1-h glucose challenge test: ≥10.0 mmol/L; 2-h oral glucose tolerance test (OGTT): ≥8.5 mmol/L (1, 4).

GDM risk factors involve obesity, family history of insulin resistance and diabetes, age, multi-parity, ethnicity, and short stature (5–7). Gestational diabetes can influence both the mother’s and the baby’s health. Preeclampsia, polyhydramnios, operative delivery, preterm delivery, and birth canal lacerations are common complications in GDM women (5, 8–10). Furthermore, the infant can develop shoulder dystocia, neonatal hypoglycemia, macrosomia (excessive fetal growth), jaundice, respiratory distress syndrome, and perinatal mortality (11–13). Women with GDM normally recovers after delivery, although both mother and child are more susceptible to developing obesity, hyperglycemia, vascular disorders, and type 2 Diabetes Mellitus (T2DM) in the future (9, 12, 14, 15).

The pathogenesis of GDM is complex and multifactorial, involving beta cell dysfunction and altered insulin secretion (10, 16). Insulin is a protein produced by the pancreas hormone that modulates the absorption and consumption of glucose (sugar) by cells. When pregnant women have insulin resistance (IR), their cells lack the ability to use insulin, and sugar accumulates in the blood (17). Additionally, increased levels of hormones such as cortisol, progesterone, placental lactogen, leptin, estrogen, among others, may also affect the body’s capacity to utilize insulin effectively during pregnancy (18). Moreover, hormonal changes can significantly affect placental function, morphogenesis, and angiogenesis. The placenta plays a vital role as the communication organ between the mother and fetus, providing necessary nutrients and oxygen. In gestational diabetes, the placental metabolism may be impacted, leading to alterations in fatty acid metabolism (19). Studies have shown that inflammatory processes can activate cytokines like IL-6, resulting in fat accumulation and inadequate fat supply to the fetus (20).

Furthermore, genetic and epigenetic changes play a significant role in placental function. Increased expression of the vascular endothelial growth factor (VEGF) and the cluster of differentiation 31 (CD31) has been observed in gestational diabetes, correlating with maternal body mass index (BMI) and weight gain. Such altered VEGF levels may lead to abnormal embryo implantation and placental formation, resulting in abnormal blood vessel growth and chronic hypoxia. Conversely, CD31 regulates inflammatory responses (21, 22). Additionally, the expression of the parathyroid hormone-related peptide (PTH-rP) and the parathyroid hormone 1 receptor (PTH-R1) in the placenta varies with maternal glucose levels. This overexpression impacts fetal growth and development through placental calcium transfer and vasodilation (23).

Epigenetic analyses have revealed high methylation levels in genes involved in the Wnt and cadherin pathways, which are critical for placental development and vascularization. Moreover, methylation of genes like IRS1, ADORA2B, and PTPRN2 (insulin regulators) has been linked to altered insulin signaling in placentas (24).

The treatment for GDM consists of controlling blood glucose levels and, most importantly, changing lifestyle through a healthy meal plan, including regular exercise. Medications, especially insulin, are administered in some cases (8). Furthermore, glucose monitoring in women with GDM can effectively regulate maternal glucose levels and maternal weight (25). However, some studies have shown that monitoring may vary among women with gestational diabetes similarly to healthy pregnant women (26).

Nutrigenomics relates diet to genes and focuses on the changes induced by nutrients in the expression of genes that control biological processes to prevent or treat some diseases (27). In the case of GDM, it has been demonstrated that environmental factors, like diet, can influence gene expression involved in pathways related to nutrient metabolism, such as beta-oxidation, ketogenesis, and the one-carbon pathway. Disruption of these pathways, including the inflammatory via, could lead to insulin resistance and raise the GDM risk (28).

Knowledge in nutrigenomics has expanded due to the emergence of new technologies such as massive sequencing, which allows a better understanding of the genome and its relationship with dietary habits (29). In this sense, nutrigenomics could be helpful for the GDM management as it grants the identification of alterations at the genomic level related to the pathogenesis of this condition and the environment.

Diet modification is the main alternative treatment for gestational diabetes. Therefore, nutrigenomics could be a therapeutic tool for GDM management by providing nutritional strategies that help the metabolic response and prevent maternal and fetal complications (30–32).

In this context, this paper aims to perform an exhaustive overview of the correlation between diet, gene expression, and the metabolic pathways related to insulin resistance to contribute to the criteria for gestational diabetes management through nutrients.

## Gestational diabetes mellitus

A pregnant woman’s body changes physiologically and anatomically to accommodate the developing fetus. Changes include weight gain, placental development, placental hormone production, and metabolic modifications (33). In the first semester of pregnancy (anabolic phase) the maternal system increases the storage of lipids in the tissues to use them as a source of energy for the last stage of gestation, during which glucose ingested by the mother is mostly used to fetal growth (33, 34). To build these energy reserves, the maternal system has to increase the energy intake and *de novo* hepatic fat synthesis (lipogenesis) (33). Increasing energy consumption requires rising insulin levels to transport sufficient blood glucose into the cells, increasing glycolysis. Additionally, elevated insulin levels suppress adipose tissue triglyceride breakdown (lipolysis), increasing lipogenesis and causing maternal white adipose tissue expansion (35–38). During the latter part of gestation, maternal glucose consumption decreases, and the maternal energy requirements are met by fatty acid oxidation; so, it is necessary to break down the adipose tissue created in the first two trimesters of pregnancy (catabolic phase) (34, 39, 40).

Glucose homeostasis is regulated by the liver throughout pregnancy. Fetus’s metabolic requirements are minimal in the early stages of pregnancy; hence, the hepatic insulin resistance is low, and the insulin levels increase. Moreover, this rise in insulin levels inhibits hepatic gluconeogenesis, causing glucose metabolism to become the primary energy production source (35, 41). In contrast, in the second half, the placental hormone production increases insulin resistance, suppressing glycolysis, elevating gluconeogenesis, and directing more glucose to the fetus. Simultaneously, during the catabolic phase, the liver also enhances fatty acid beta-oxidation (lipolysis) (41). Despite high glucose and fat levels at this stage, most pregnancies have normal glucose ranges due to the maternal pancreas production and secretion of more insulin (42).

In summary, in the final stretch of a normal pregnancy, the maternal system overcomes insulin resistance and reaches glucose homeostasis by elevating the amounts of plasma insulin (43). However, in some cases, severe insulin resistance causes impaired pancreatic beta cells, leading to GDM (40, 44–47). Kim et al. (48) suggested that pancreatic beta cells increase insulin secretion in response to specific hormonal and metabolic signals to maintain facilitated anabolism.

Furthermore, there are not only physiological or anatomical processes in gestational diabetes but also changes at the cellular level involving pathways specifically related to the production of molecules that lead to insulin resistance or hypoglycemia, such as ketone bodies, oxaloacetate, and glucose, among others (44).

## Molecular pathways in GDM associated with insulin resistance

Some of the more important metabolic pathways altered in GDM, which coincide with the dysregulated pathways in T2DM, are glycolysis, beta-oxidation, and metabolism of ketone body, asparagine, and one-carbon. In addition, inflammation mediated by the Toll-like receptor 4/Nuclear factor kappa B (TLR4/NF-κB) signaling pathway is essential in GDM development.

### Glycolysis, beta-oxidation, ketone bodies, and asparagine metabolic pathways

In the human body, when insulin binds to its receptor (IRe), insulin receptor substrate (IRS-1 and IRS-2) is activated by phosphorylation. Phosphorylated IRSs activate two intracellular pathways: (1) the mitogen-activated kinase (MAPK) cascade, which promotes the expression of several proteins, including glucose transporter 1 and 4 (GLUT1 and GLUT4). (2) the phosphatidyl-inositol 3-kinase (PI3K) which enhances the GLUT vesicles translocation to the plasma membrane. Importantly, GLUT proteins are responsible of the glucose transport into the cells (49, 50).

Otherwise, during a healthy pregnancy, the increase in plasma fatty acids concentration causes a decrease in intracellular glucose levels, indicating that fatty acids disrupt insulin-induced glucose transport activity. These changes were related with PI3K activity reduction and decreased tyrosine phosphorylation of IRS-1 (51).

Thus, due to the increment of plasma fatty acid levels, it is necessary to regulate their metabolism. Several studies have established that a higher discharge of fatty acids into the mother’s bloodstream leads to increased carnitine use, reducing its concentration in the circulation (52, 53). Carnitine is an indispensable metabolite in energy production because transports fatty acids into the mitochondria for beta-oxidation and subsequent ketone bodies formation or Adenosine triphosphate (ATP) generation through the tricarboxylic acid (TCA) cycle (54). In addition, reduced levels of carnitine also could be produced by increased uptake of carnitine from the maternal bloodstream to the placenta for normal fetal development (55, 56). Huo et al. and Dudzik et al. indicated that serum carnitine concentration was reduced in pregnancies with GDM, hence, carnitine deficiency may lead to impaired lipolysis (57–60).

Moreover, fatty acids transport into the mitochondria is carried out by three proteins: carnitine-acylcarnitine translocase (CACT), carnitine palmitoyltransferase I and II (CPT I and CPT II). CPT I uses free carnitine from the cell to convert acyl-CoA into acylcarnitine. The acylcarnitine is transported to the mitochondrial matrix by CACT, then, it is reconverted to acyl-CoA by CPT II. The released carnitine diffuses into the cytoplasm and can be reused by CPT I (61).

Concurrently, in the mitochondrial matrix of hepatocytes, the acetyl-CoA becomes a substrate for the ketogenesis or combines with oxalacetate to be metabolized in the TCA cycle. Elevated production of ketone bodies is caused because much of the oxaloacetate is converted to glucose, with a small proportion remaining to enter in the TCA cycle. A decrease in oxaloacetate, is a characteristic event during GDM (33, 60).

The elevated synthesis of ketone bodies is exacerbated in states of excessive acetyl-CoA synthesis from fatty acids. These states may occur due to frequent alcohol consumption, reduced carbohydrate intake, and insulin resistance. Several studies have associated low oxaloacetate availability with high levels of ketone bodies in women with GDM at different stages of gestation (60, 62–65).

**Figure 1:**
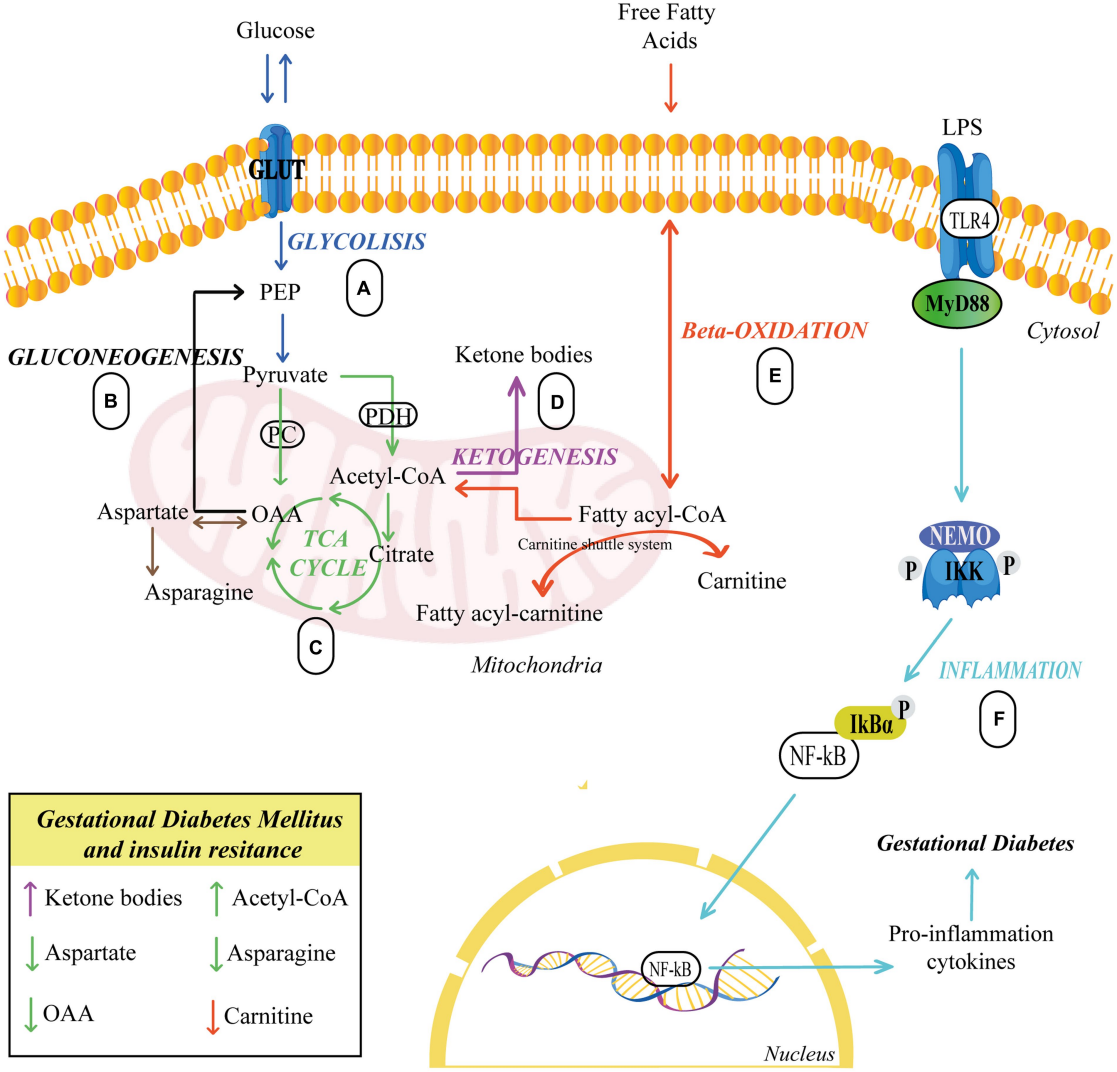
Pathways related to insulin resistance. **(A,C)** Glucose breaks down producing pyruvate, which serves to produce energy in the TCA. **(B)** Glucose biosynthesis occurs from oxaloacetate. **(C–E)** Carnitine carries fatty acids into the mitochondria to synthesize acetyl-CoA, which serves to generate energy in the TCA cycle or to form ketone bodies via ketogenesis. **(F)** TLR4 activation sets off a series of events that culminate in the activation of NF-B, which is responsible for the synthesis and release of proinflammatory cytokines, eventually leading to GDM. Upward arrows indicate an elevation, while downward arrows indicate a reduction. Different colors represent each cellular process. GLUT, Glucose Transporter; PC, Pyruvate carboxylase; PDH, Pyruvate dehydrogenase; PEP, Phosphoenolpyruvate; OAA, Oxaloacetate; LPS, lipopolysaccharide; TLR4, Toll-like receptor 4; IKK, IκB Kinase; NF-kB, Nuclear factor κB.

Finally, several authors have described reduced concentrations of aspartate in women with gestational diabetes, which could signal the forced production of oxaloacetate by the cell. Additionally, significantly lower levels of asparagine were also found, resulting from a probable deficiency of oxaloacetate and aspartate. Reduced asparagine levels have been linked to the occurrence of T2DM in non-pregnant women (66). Figure 1 illustrates the alteration in glycolysis, beta-oxidation, and the metabolism of ketone body and asparagine in GDM.

### One-carbon metabolic pathways

**Figure 2:**
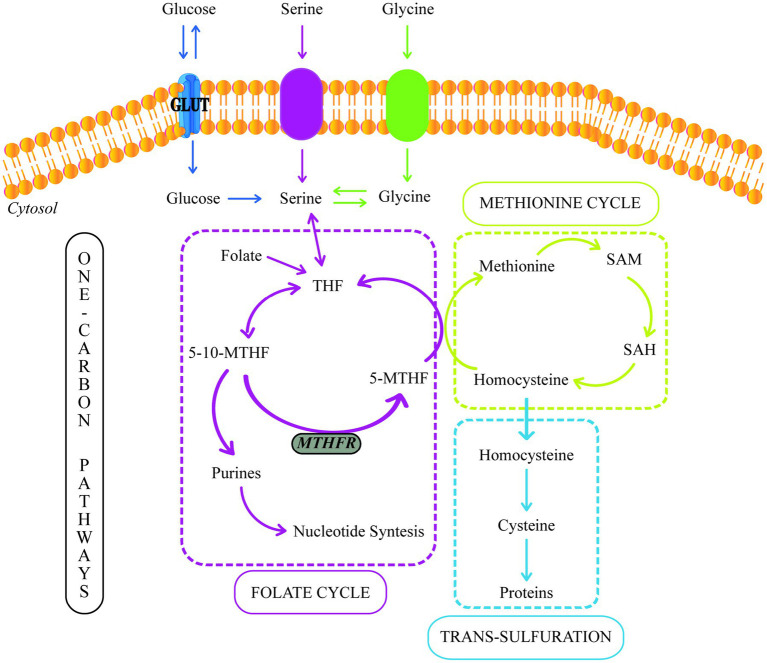
One-carbon metabolism. GLUT, Glucose Transporter; THF, Tetrahydrofolate; 5-10-MTHF, 5,10-methylenetetrahydrofolate; 5-MTHF, 5-methyltetrahydrofolate; MTHFR, methylenetetrahydrofolate reductase; SAM, S-adenosylmethionine; SAH, S-adenosylhomocysteine.

The term one carbon (1C) is used to refer to functional groups containing only one carbon. In this context, 1C metabolism involves the methionine and folate cycles and the transsulfuration pathway and requires many 1C units (64) (Figure 2). 1C metabolism is fundamental in various cellular processes, such as amino acid homeostasis, redox defense, and methylation reactions (67). In a healthy pregnancy, there are deficiencies in 1C metabolism during intrauterine fetus development (68, 69). However, several studies have shown that in women with GDM, the levels of serine, glycine, methionine, and histidine (1C units) decrease even more.

In the cytosol and mitochondrion, 1C units could be supplied by the conversion of serine to glycine, catalyzed by serine hydroxymethyltransferase (SHMT). The placental SHMT is indispensable for meeting the glycine requirements of the fetus during its development. Moreover, numerous studies have reported a glycine decrease in patients with T2DM and women with GDM (60, 67, 70).

Additionally, GDM is also related to increased oxidative stress, which increases glutathione biosynthesis due to the extended antioxidant placenta capabilities to minimize oxidative damage (71). Spanou et al. (72) discovered lower levels of glycine and glutamate in GDM women. Their study supports the hypothesis of increased glutathione synthesis since glycine and glutamate are its precursors. Furthermore, glycine is a substrate for gluconeogenesis; thus, decreased glycine levels during GDM may indicate an enhanced activity of this process (60, 72).

During the glycine synthesis from serine, the 1C transfer produces 5,10-methylenetetrahydrofolate (5,10-MTHF). Moreover, 5,10-MTHF could be reduced to 5-methyltetrahydrofolate (5-MTHF) by the methylenetetrahydrofolate reductase (MTHFR). 5-MTHF is the active form of folate, the most common circulating form of folate, and a methyl donor for homocysteine remethylation to methionine.

Folic acid (FA) metabolism is part of the folate cycle. In this sense, the enzyme dihydrofolate reductase (DHFR) is required to catalyze FA’s conversion to tetrahydrofolate (THF) (73). Then, THF is metabolized to 5-methylTHF via the one-carbon metabolic pathway by methylenetetrahydrofolate reductase (MTHFR). Thus, MTHFR is critical in regulating folate availability (74).

On the other hand, methionine can also be used as a source of 1C units due to its conversion to S-adenosylmethionine (SAM), a reactive methyl carrier implicated in a variety of cellular functions (75). Furthermore, methionine, threonine, and cystathionine metabolism can produce 2-ketobutyrate (2-KB), which can be metabolized to 2-hydroxybutyrate (2-HB) or 2-aminobutyrate (2-AB) (76, 77). 2-HB is a compound that increases during oxidative stress (76). Many studies have suggested that 2-HB is an indicator of insulin resistance and defective glucose metabolism (65, 78, 79). Spanou et al. (72) discovered elevated levels of 2-HB and lower levels of 2-AB in women with GDM. These findings point to a predilection for the formation of 2-HB rather than 2-AB due to enhanced lipid oxidation and oxidative stress, which are the results of increased circulating free radicals or a disruption in the antioxidant systems (72, 80).

### TLR4/NF-κB signaling pathway

Toll-like receptor 4 (TLR4) is a transmembrane glycoprotein, a member of the Toll-like receptor (TLR) family. TLR4 plays an essential role in recognizing pathogen-associated molecular patterns (PAMP) and activating the innate immunity response (81). Moreover, TLR4 consists of two domains: an internal TIR (Toll-interleukin-1 receptor) and an external LRR (leucine-rich repeat). When a PAMP is recognized, TLR4 forms a complex with other proteins. This oligomerization leads to conformational changes, which, in turn, trigger the recruitment of intracellular adaptor proteins through protein–protein interactions (82). There are five known TIR domain-containing adaptor proteins: MyD88 (myeloid differentiation primary response gene 88), TIRAP (TIR domain-containing adaptor protein), TRIF (TIR domain-containing adaptor inducing IFN-b), TRAM (TRIF-related adaptor molecule), and SARM (sterile alpha and HEAT-Armadillo motifs-containing protein) (82–84).

TLRs use several adaptor protein combinations, each of which triggers a specific intracellular signaling cascade that activates the innate immune response (84). TLR4 stimulation promotes two metabolic pathways: the MyD88-dependent or the MyD88-independent. In the MyD88-dependent pathway, MyD88 phosphorylates and ubiquitinates multiple signaling proteins, leading to the activation of NF-κB and MAPKs. Subsequently, NF-κB translocates to the nucleus and regulates the production of proinflammatory cytokines, including tumor necrosis factor-alpha (TNF-α), interleukin 1 (IL-1), and interleukin 6 (IL-6) (82). In the MyD88-independent pathway, TRIF stimulates the transcriptional factor IRF3 (interferon regulatory factor 3), triggering the synthesis of type I interferon (IFN-I) and the activation of IFN-I-inducible genes. Furthermore, it activates NF-κB and MAPKs at later stages (82, 85).

The increased expression and activation of the TLR4/MyD88/NF-kB pathway in the placenta lead to an enhanced synthesis and release of inflammatory cytokines and bio-mediators. This, in turn, may affect insulin signaling and conduce to severe insulin sensitivity impairment in the placentas of women with GDM (86). Figure 1 represents the inflammation pathway in GDM.

Insulin resistance develops due to increased levels of cytokines and hormones that inhibit insulin signaling (87). IRS-1 and Akt are essential components of the PI3K insulin signaling cascade (88). An increase in the phosphorylation of IRS-1 serine residues disrupts various biological processes, including IRe function, phosphorylation of IRS-1 tyrosine residues, and PI3K pathway activity, resulting in reduced glucose absorption (89). Furthermore, Akt phosphorylation allows GLUT4 translocation to the plasma membrane. For instance, Feng et al. (86) observed that the TLR4/MyD88/NF-κB pathways were overexpressed in the placenta of women with GDM, suggesting a potential role in insulin resistance. Moreover, Mrizak et al. (90) explored the role of pro-inflammatory factors in the placenta of women with GDM, and the results corroborate that the mRNA expression of the TLR4 pro-inflammatory factor increased in the GDM placenta.

## Nutritional genomic patterns and molecular pathways in gestational diabetes mellitus

The understanding of nutritional genomics could aid the development of diets to avoid and treat illnesses. In this sense, lifestyle changes are critical in gestational diabetes management. Diet is a modifiable environmental factor that constitutes the first step in GDM treatment. A healthy diet consists of a balance of macronutrients (protein, carbohydrates, and fats) and micronutrients (vitamins and minerals) for normal physiologic functioning (91); however, dietary patterns differ according to cultural, individual, environmental, economic, familial, food availability, and other factors (92, 93).

The interaction between genes and nutrients may be essential in determining the phenotype associated with diseases like gestational diabetes (94). Several studies have revealed that dietary patterns and gene expression related to molecular pathways may also be risk factors for diabetes, especially type 2 and gestational (95–97).

In this section of our review, associations between dietary patterns are established, including nutrients, gene expression, and molecular pathways with GDM. Also, studies of T2DM are included due to gestational diabetes could predispose to developing diabetes type 2 after delivery.

### Carbohydrates and GDM

Several investigations suggest that pregnant women need about 175 g of carbohydrates per day (47% of total calories) (98, 99). A study analyzed the effect of low-carbohydrate diet in women with GDM and discovered that women with less than 42% carbohydrate consumption had less health problems than women with more than 45% carbohydrate intake. Furthermore, they found that proper glycemic control reduced the requirement for insulin therapy, fewer cesarean deliveries, and less incidence of macrosomia and large-for-gestational-age babies (100). In contrast, Radesky et al. (101) analyzed 297 women who developed GDM and had glucose intolerance. They observed that nutrient intake (saturated fats, polyunsaturated fats, and carbohydrates) in early pregnancy showed no relationship with the risk of GDM (101).

On the other hand, numerous investigations have suggested that a diet low in carbohydrates during pregnancy may increase urinary ketone levels (ketonuria) (102–104). Ketonuria is caused by inefficient glucose utilization, and cells metabolize lipids to obtain energy. These ketones can cross the placenta and harm the baby’s cognitive development; as a result, various studies disagree on the benefits of a low-carbohydrate diet (102, 103, 105). However, one study explored 46 women with GDM on a low-carbohydrate diet (approximately 135 g/day) and found that lowering carbohydrate intake did not significantly increase ketones. This implies that more research on the relationship between diet and this condition is required to assess the benefits and risks (106).

Consequently, understanding carbohydrate metabolism is fundamental in the GDM management. In the digestive tract, carbohydrates ingested by the maternal diet are broken down mainly into glucose, which is transported into the bloodstream and used as a source of energy by the mother and the developing fetus through the placenta (107). Furthermore, blood glucose also comes from hepatic gluconeogenesis and glycogen metabolism. In the mother, the glucose is transported to pancreatic beta cells, where it enters the carboxylic acid cycle within the mitochondria, producing energy in the form of ATP. The rise in cytosolic ATP leads to insulin exocytosis (108, 109). Therefore, insulin plays an essential function in the control of glucose metabolism in the liver, muscle, and adipose cells.

In the second period of pregnancy, maternal tissue cells become insulin resistant to provide more glucose for fetal development (34, 41). Insulin resistance is a natural process that is reversed after delivery (110). Therefore, it leads to the activation of several compensatory mechanisms by the maternal system to maintain glucose homeostasis during this stage. In a healthy gestation, the main compensatory mechanism is the increase of circulating insulin levels by the maternal pancreas (43). However, alterations in this mechanism could result in hyperglycemia and excessive endogenous glucose production by the liver (34, 44). Thus, prolonged exposure to hyperglycemia (glucotoxicity) may result in beta cell dysfunction and reduce insulin secretion, which could be permanent (111). In summary, GDM would be triggered by impaired pancreatic beta cell function (108, 109).

Nutrients have significant effects on beta cells (112, 113). For this reason, the regulation of carbohydrate intake plays an essential role in blood sugar level control. Glucose enters the cells through the glucose transport facilitator systems (GLUT), these transporters are expressed in all tissues of the organism, constituting the main mechanism of glucose entry into all cells. One of the most studied transporters is GLUT4 because it has an elevated affinity for glucose and is expressed in tissues susceptible to insulin. GLUT4 is localized in the cytoplasm and stored into vesicles. Then, the vesicles are translocated to the plasma membrane, and this process is controlled by insulin (49). In addition, IRS is a key modulator of insulin signal activation, which is essential for the transfer of GLUT4 and glucose transport (114).

Krause et al. (115) treated HepG2 cells with high concentrations of insulin and glucose and observed that hepatic let-7e-5p miRNA expression was increased, and IRS-2 protein expression was decreased. The group of researchers suggested that let-7e-5p miRNA is very important in modulating hepatic IRS-2 expression (115).

Moreover, Ide et al. (116) evaluated the level of expression of SREBP-1, IRS-1, and IRS-2 in the liver of sucrose-rich diet mice. The authors determined increased levels of SREBP-1 and decreased levels of IRS-2, compared to the levels of the same proteins in fasting mice. They also observed that IRS-1 levels were not significantly altered, suggesting that SREBP-1 may regulate IRS-2 gene expression (116).

The specific causes of the alterations in the compensatory processes of the pancreatic beta cells in GDM have not been certain determined; however, it is likely that this failure is multifactorial and involves variations in genes and protein expression caused by nutrient overload, inducing oxidative stress, or elevated inflammation (47).

Accordingly, the intake of carbohydrate-rich foods primarily influences the expression of IRS and GLUT genes, which are involved in the glycolysis pathway.

### Nutrients from fruits and vegetables in GDM

Fruits and vegetables are nutritionally essential in a healthy diet because they are rich in vitamins, minerals, and antioxidants (such as flavonoids, anthocyanins, and polyphenols). Their high fiber and antioxidant content can help reduce inflammation and prevent disease (117). According to the American Diabetes Association (ADA), women with GDM should consume 28 g of fiber, which means that they should eat 600 g of fruits and vegetables per day, with a minimum of 300 g of vegetables, focusing on fibrous and rough vegetables (118, 119).

Fruits and vegetables rich in vitamins, antioxidants, or FA are recommended during pregnancy for the baby’s development and healthy weight. However, some fruits contain a lot of sugar and should be avoided during pregnancy. Low glycemic index fruits, such as cherries, prunes, grapefruit, dried apricots, raisins, peaches, apples, pears, strawberries, plums, guava, oranges, grapes, papaya, bananas, kiwi, pineapple, figs, and mangoes, are recommended to be consumed by diabetic pregnant women (120). The increase in blood glucose by high glycemic index fruits can cause inflammation, which is more evident in obese pregnant women (121).

A Chinese population case-control study (1,464 GDM women and 8,092 healthy women) examined dietary patterns and the risk of gestational diabetes. The vegetable dietary pattern was defined by the consumption of vegetables like carrots, tomatoes, Chinese greens, cabbage, eggplants, mushrooms, potatoes, peppers, bamboo shoots, agaric, garlic, and bean products. They found that eating vegetables reduced the risk of developing GDM by 6–9% (122). Furthermore, Asadi et al. (123) found a negative association with the risk of the GDM development, and a dietary pattern based on a higher intake of fruits, low-fat dairy, potatoes, eggs, poultry, fish, nuts, offal, and red meat. Also, a case-control study analyzed 460 pregnant women and discovered that a plant-based diet was correlated with a reduced risk of GDM (124).

Consequently, diets with an elevated consumption of vegetables and fruits are the most recommended for the prevention and treatment of GDM. Several investigations have focused on analyzing the compounds or active principles of fruits or vegetables to improve insulin resistance (125). Flavonoids, anthocyanins, polyphenols, among others have been tested in rat and mouse models to measure gene expression levels of pathways that regulate insulin resistance (126, 127).

Therefore, increasing the consumption of low glycemic index fruits but rich in antioxidants, such as flavonoids, may help to decrease inflammation mediated by the TLR4/NF-B signaling pathway. For instance, Duan et al. (128) investigated the anti-inflammatory effects of flavonoids in a rat model with T2DM. After 8 weeks of using flavonoids, they found that plasma glucose and insulin resistance decreased. In addition, they found that the liver inflammation of T2DM rats significantly improved, due to the expression of myeloid differentiation factor 88 (MyD88), TNF receptor-associated factor 6 (TRAF6), toll-like receptor 4 (TLR4), an inhibitor of NF-κB alpha (IκΒα), p-IκΒα, and NF-κB were downregulated (128). Other studies found that anthocyanins reduced insulin resistance by inhibiting hepatic inflammation through the reduction of TLR4/NF-κB/JNK in liver tissues and improving oxidative stress. TLR4-mediated JNK and NF-κB inflammatory vias inhibit insulin signaling in the liver (129).

Moreover, Kim et al. (130) treated pancreatic beta cells (RINm5F) with mixtures of inflammatory cytokines, with or without flavonoids. As a result, the flavonoids decreased cytotoxicity in cells and mitigated the decrease of glucose-stimulated insulin production due to inhibiting the nitric oxide synthase (NOS) gene expression. Naringin, another bioflavonoid, was tested in rats with T2DM (caused by streptozotocin and a high-fat diet) for insulin resistance improvement and beta cell dysfunction. The authors found that naringin improves insulin resistance by regulating oxidative stress, inflammatory processes, and upregulation of peroxisome proliferator-activated receptor gamma (PPARγ) and heat shock protein-27 and 72 (131).

Similarly, other studies have been conducted with the polyphenol epigallocatechin gallate (EGCG) to evaluate its antioxidant and protective properties on pancreatic beta cells. EGCG can be found in fruits, vegetables, cocoa-based products, and green teas (132). Zhang et al. (133) evaluated the effects of EGCG on beta cells by exposing insulin-producing cell lines, with and without EGCG pretreatment, to a combination of citokines like interleukins and among others. The researchers found that EGCG protects against cytokines, restores insulin secretion, and prevents cytokine-induced iNOS overexpression (133).

Furthermore, another study analyzed the effect of EGCG and rutin. The antioxidant rutin comes from the triffid of buckwheat (*Fagopyrum esculentum*) and has been studied in diabetic rat models due to its ability to decrease blood glucose levels. The investigation group found that Rutin and EGCG have anti-diabetic benefits by providing pancreatic beta cell protection in rats by attenuating the glucotoxicity effects through activation of AMPK to inhibit NF-κB-induced inflammatory responses (134).

These studies show that a diet rich in vegetables and fruits can be used for the GDM management, due to the relationship between antioxidants present in these, gene expression, and reduction of inflammation (induced by the activation of the TLR4/NF-κB pathways).

### Fats in GDM

According to the ADA, pregnant women’s diets should be low in saturated fats (128). Meanwhile, the Institute of Medicine recommends that 25-40% of energy come from fats (135). A study of 1,698 pregnant women’s dietary intake found a correlation between an risen fat consumption and the development of glucose imbalances during the second trimester of pregnancy (136). Consequently, high-fat consumption should be avoided in GDM because it is associated with infantile adiposity, maternal inflammation, and placental dysfunction (119).

However, some fats play a vital function in the growth and development of a baby throughout pregnancy. For instance, unsaturated fats like polyunsaturated fatty acids (PUFAs) are a type of healthy fats found in fish oils, vegetable oils, and some nuts. Pregnant women should consume a minimum of 350 g of fish per week, of which 200 g should be fatty fish (anchovies, black cod, salmon, sardines, bluefin tuna, and others) (119, 137, 138).

An investigation discovered a correlation between saturated and polyunsaturated fats consumption, and gestational diabetes in patients with and without risk factors (older, shorter in stature, significantly higher BMI before pregnancy, and a high percentage of diabetic first-degree relatives). They found an increased risk of developing GDM when consuming saturated fats (139).

Thus, controlling fat intake is fundamental in the GDM management. Fatty acid metabolism is mediated by beta-oxidation and ketogenesis and may have effects on gene expression in pancreatic beta cells and other cells (140). A study established that CPT I activity is key in the control of fatty acid oxidation (141). Bruce et al. (142) concluded that increased CPT I expression promotes beta-oxidation of fatty acids, ameliorating the effect of high-fat diet-induced insulin resistance.

Another study in healthy mice evaluated *CREB3L3*, *PPARα*, *CPT I*, and *BDH1* genes due to their essential role in regulating fatty acid oxidation, fatty acid transport and ketogenesis. Nakagawa et al. (143) analyzed mice on a ketogenic diet and found that Creb3l3^−/−^ mice exhibited reduced expression in fat oxidation and ketogenesis genes such as *CPT I* and *BDH1* (D-β-hydroxybutyrate dehydrogenase). Thus, they found that reduced expression of *CPT I* in the liver of Creb3l3^−/−^ mice inhibited fatty acid transport into mitochondria and suppressed fatty acid oxidation. Moreover, *BDH1*, a ketogenesis gene that together with HMGCS2 and HMGCL enzymes, transforms acetyl-CoA to β-OH butyrate, was up-regulated by CREB3L3, altering the ketogenesis. Finally, they found in Pparα^−/−^ mice a decrease in the oxidation of other fatty acids, suggesting that CREB3L3 cooperates with PPARα, directly and indirectly, by modulating the expression of genes involved in the metabolism of fatty acids and ketogenesis (143).

Besides, Radler et al. (144) studied the PPAR pathways and determined that consumption of PUFAs, polyphenols, and L-carnitine increased the amount of peroxisome proliferator-activated receptor (PPARα) mRNA and its target genes (CPT I, carnitine acetyltransferase and organic cation transporter 2) were also upregulated in cells of the peripheral blood.

Consequently, carnitine, PUFAs, antioxidants, and polyphenols could help to reduce the deposition of partially degraded long-chain fatty acids and be useful in GDM treatment and prevention.

## Discussion

Gestational diabetes is one of the most prevalent medical issues during gestation, and its associated conditions can further complicate the disease, including the offspring’s health. Different studies have found a correlation between gestational diabetes and insulin resistance. Ryan and collaborators investigated the mechanisms of insulin resistance by measuring glucose infusion rates in non-pregnant (213 ± 11 mg/m^2^ • min), pregnant without GDM (143 ± 23 mg/m^2^ • min), and pregnant with GDM (57 ± 18 mg/m^2^ • min). They found that pregnant women with gestational diabetes had more evident insulin resistance (145).

Another study analyzed pregnant women with GDM and the presence or absence of insulin resistance. They found that all women showed impaired pancreatic beta cell function. Moreover, they observed that GDM with increased insulin resistance has an elevated risk of adverse pregnancy outcomes and complications like hyperglycemia, elevated BMI, blood sugar, and lipid levels (146).

The ADA recommends that women with gestational diabetes have access an individualized nutrition plan to avoid the complications caused by insulin resistance. The diet recommended should include a balance of micronutrients and macronutrients to maintain fetal growth while limiting postprandial glucose excursions and maintaining normal gestational weight (17, 119). However, different studies contradict each other regarding the intake percentages of each macronutrient or micronutrient; but some of them agree that the diet should have an increased intake of fruits, vegetables, and fish and a reduction to a minimum of processed foods, artificial sweeteners, sugary drinks, sweets, and high glycemic index foods (136, 147, 148). He et al. (97) studied the correlation between different dietary patterns and GDM development. They found that the vegetable diet pattern was associated with a lower risk of gestational diabetes while the sweets and seafood diet pattern was correlated with a higher incidence of GDM, concluding that the vegetable diet pattern had a more evident protective effect in women with a familial diabetes (97).

Furthermore, a healthy diet before pregnancy may help to prevent GDM and IR. A study monitored the diet of pregnant women 1 year before pregnancy. They discovered that the Mediterranean diet, high in vegetables, fruits, cereals, legumes, fish, dairy products, and lesser extent meat, had a protective effect against gestational diabetes (149).

On the other hand, different research groups evaluated the correlation between ingested nutrients and gene expression in different tissues and cells. As a result, they have found that gene expression changes, disrupt physiological cell mechanisms and increase or reduce GDM risk. In addition, the use of cell lines or mouse models has facilitated the investigation of gene expression and diet-induced beta cell dysfunction (150, 151). For example, Koloverou et al. (152) found that the Mediterranean diet is high in anti-inflammatory compounds. Mediterranean diet has an anti-diabetic effect because attenuates the inflammatory state using multiple mechanisms, such as the reduction of oxidative stress and the regulation of NF-κB and PPARγ pathways.

Other studies have evaluated low-fat plant foods such as curcuma and soy. These foods were studied for their antioxidant properties because they may also help regulate gene expression in beta-oxidation. Lone et al. (153) demonstrated that curcumin produced by curcuma, upregulates CPT I expression in brown adipocytes. Likewise, Meléndez-Salcido et al. (154) established that curcumin induces CPT I overexpression in cardiac tissue. Another study concluded that physical training and soy protein intake play a fundamental role in the induction of PPAR pathways, leading to increased CPT I enzyme activity and also increased mRNA levels of CPT I and other enzymes involved in lipid oxidation in muscle (155).

Also, several studies have established that excess lipids cause or worsen insulin resistance through multiple mechanisms (156). Ringseis et al. (157) discovered that a significant amount of partially metabolized fatty acids in muscle caused mitochondrial stress, inhibiting both insulin signaling and glucose metabolism. Moreover, high-fat diet studies found variations in the expression of other genes. For instance, Qiu et al. (158) analyzed normal and T2DM mice induced by a high-fat diet. They found that REG1, REG2, and GSHPX1 were differentially expressed in the pancreas of normal mice and mice with diabetes. In the diabetic mouse model, up-regulation of the three proteins increased pancreatic beta cell proliferation and insulin production (158). Another altered protein in pancreatic cells is WNT4, reported by Kurita et al. (159). They evaluated mice with a diet rich in fats and sucrose and discovered over-expression of WNT4 in beta cells, which resulted in enhanced glucose-induced insulin production and reduced beta cell proliferation (159).

Other investigations have been focused on the gene expression that could alter the pancreatic beta cell function. For instance, Hall and collaborators evaluated the effect of elevated glucose concentrations on gene expression in human pancreatic islet cells. The researchers exposed cells to a high glucose level (19 mM) and a control concentration (5.6 mM). After 48 h, they determined that exposure to 19 mM glucose significantly altered the expression of *SLCO5A1*, *RASD1*, *SYT16*, *GLRA1*, *TMED3*, *VAC14*, *CHRNA5*, and *LEPREL2* genes (160).

Liu et al. (161) determined the effects of glucotoxicity on gene expression in pancreatic islet microvascular endothelial cells (IMECs) by treating them with 5.6 mmol L-1 glucose (control group), 35 mmol L-1 glucose (glucotoxicity), and 35 mmol L-1 glucose plus 10-8 mol L-1 insulin. The study group determined that glucotoxicity resulted in the differential expression of 1,574 mRNAs compared to the control and 2,870 mRNAs relative to the insulin-treated group. In addition, they also identified that these deregulated genes played roles in the regulation of proliferation, apoptosis, adhesion, migration, and metabolic activities (161).

While alterations at the mRNA level can affect a variety of cellular and molecular processes, there are compensatory mechanisms at the translational level that allow dynamic regulation of gene expression in response to changes in the cellular environment, intracellular signals, and adaptation to various stressors (162, 163).

Furthermore, Nakane et al. (164) used rat insulinoma INS-1 cells as a model of pancreatic glucotoxicity and treated them with 11.2 mM and 22.4 mM glucose for 7 days. The scientists found that insulin gene expression increases with 11.2 mM of glucose and decreases with 22.4 mM. Additionally, they also determined that the expression of protein kinase CPG16 increased, pointing to its importance in suppressing insulin gene expression in pancreatic beta cells under cytotoxicity (164).

Impaired lipolysis and glucose homeostasis could be controlled by nutrients obtained from the diet, thus preventing the development of GDM. However, several studies have established the need for nutritional supplement consumption, such as folic acid, because they are difficult to obtain from the diet alone. Furthermore, supplements could also influence gene expression (165, 166).

The Centers for Disease Control and Prevention (CDC) suggests that women of reproductive age who plan to become pregnant consume at least 400 ug of folic acid per day. A woman can obtain the folate needed for pregnancy by following a balanced diet rich in natural folate or taking vitamins containing folic acid (165). Folic acid prevents neural tube defects, and many studies have established recommendations for FA supplementation before and during pregnancy (167). However, excessive intake may be associated with the development of GDM (168–170). Researchers evaluated folic acid as a dietary supplement in pregnant mice and rats and found that high doses of FA (40 mg/kg) during gestation caused glucose intolerance and insulin resistance (171, 172). Moreover, Reynolds (173) and Kelly et al. (174) found that doses larger than 260-280 g of FA lead to circulating unmetabolized folic acid (uFA) as a result of DHFR capacity saturation. High intake of FA during the periconceptional period (approximately 1,000 μg/day) results in elevated uFA levels in pregnancy (173, 174).

Several studies have also suggested that excess FA causes inhibition of folate-dependent pathways, promoting the thymidylate synthase (TS) process rather than the methionine synthase (MTR) cycle. MTR activity is directly proportional to vitamin B12 availability and is necessary to support homocysteine remethylation processes (175). Koseki et al. (176) and Ortbauer et al. (177) demonstrated that elevated FA content in *C. elegans* causes a decrease in the expression of methylenetetrahydrofolate reductase (MTHF-1), methionine synthase (METR-1) and methionine synthase reductase (MTRR-1); and an increase in TS expression. Elucidating the mechanism by which the organism favors TS activity over MTR activity is essential because many investigations have proposed elevated folate and reduced vitamin B12 interaction during gestational diabetes (176, 177).

In addition, studies in mice have suggested that high dietary FA intake induces a functional alteration in MTHFR. Bahous et al. (178) determined that a FA intake 10 times higher than recommended could reduce the concentration of MTHFR protein in the liver of pregnant mice. Additionally, the same study indicates that a FA intake 5 times higher than recommended may decrease the amount and activity of MTFR protein in the maternal liver (178).

Regarding iron supplementation for preventing anemia during pregnancy, several studies have raised concerns about a potential association between high iron levels and an increased risk of gestational diabetes (179, 180). However, contradictory findings have been reported, with some researchers finding no adverse effects of iron supplementation on pregnant women (181, 182). Nevertheless, it is crucial to monitor iron levels before considering it as a supplement due to its potential impact on glucose metabolism and insulin resistance (183).

On the other hand, probiotics have also shown potential benefits for glycemic control in women with GDM. A meta-analysis indicated that probiotic supplementation can improve blood sugar levels and positively influence other metabolic parameters, including insulin sensitivity and lipid profiles (184). However, further research is needed to fully understand the mechanisms underlying these effects and to determine the most effective probiotic strains and dosages for GDM treatment.

GDM is a complex disease in which the patient’s genetic background and environment must be considered. Without a doubt, balanced nutrition may be beneficial in GDM management; in addition, knowing the genes related to GDM and determining what kind of food can positively interact with them and be helpful in the treatment and management of this disease. In this review, we show several studies that pointed to the interaction of the active ingredients of certain foods with genes related to glucose and fatty acid metabolism, oxidative stress, and control of insulin secretion, among others. Most of these studies have determined a change in the expression level of these genes.

In conclusion, our review supports that the relationship between nutrients and gene expression could alter metabolic pathways that trigger insulin resistance in GDM. Thus, nutritional advice and physical exercise are critical for improving the health of mothers and their babies, especially for women who are overweight or have other associated risks for gestational diabetes mellitus.

## Author contributions

PG-R, EP-C, and AZ: conceptualization. AZ and DS-R: resources and supervision. EP-C, PG-R, VR-P, AZ, SC-U, RT-T, MF, and DS-R: writing—review and editing. AZ: project administration. AZ and DS-R: funding acquisition. All authors contributed to the article and approved the submitted version.

## Funding

The publication fee of this article is funded by Universidad UTE.

## Conflict of interest

The authors declare that the research was conducted in the absence of any commercial or financial relationships that could be construed as a potential conflict of interest.

## Publisher’s note

All claims expressed in this article are solely those of the authors and do not necessarily represent those of their affiliated organizations, or those of the publisher, the editors and the reviewers. Any product that may be evaluated in this article, or claim that may be made by its manufacturer, is not guaranteed or endorsed by the publisher.
